# Psychosocial predictors of emotional eating among Thai nurses: a cross-sectional study

**DOI:** 10.1186/s12912-025-04086-6

**Published:** 2025-11-24

**Authors:** Siripan Naknoi, Krisada Suamchaiyaphum, Sasithorn Tomon

**Affiliations:** Princess Agrarajakumari Faculty of Nursing, Chulabhorn Royal Academy, Bangkok, 10210 Thailand

**Keywords:** Burnout, Emotional eating, Nurses, Psychosocial, Thailand

## Abstract

**Background:**

Nurses face high levels of stress and burnout that contribute to unhealthy coping behaviors, including emotional eating. While studies have examined psychosocial correlates of emotional eating, evidence remains limited in Thailand. This study aimed to investigate the association between psychosocial factors and emotional eating among Thai nurses.

**Methods:**

A cross-sectional survey was conducted among 168 nurses working across Thailand between January and May 2025. Data were collected using validated instruments, including the Jenkins Sleep Questionnaire, Copenhagen Burnout Inventory, Generalized Anxiety Disorder-7, Patient Health Questionnaire-9, and the Emotional Eating Questionnaire. Multiple linear regression was used to identify psychosocial predictors.

**Results:**

The model explained 33% of the variance in emotional eating. Client-related burnout (B = 0.054, *p* = 0.007), anxiety (B = 0.526, *p* < 0.001), and depression (B = 0.233, *p* = 0.034) were positively associated with emotional eating, while personal burnout was negatively associated (B = -0.057, *p* = 0.009). Sleep problems and work-related burnout were not found to be significant.

**Conclusions:**

Personal burnout showed an inverse relationship, highlighting the complex and domain-specific nature of burnout in relation to coping behaviors. Interventions should target the reduction of client-related stress and psychological distress, alongside resilience-building strategies, to mitigate emotional eating and promote the well-being of nurses.

## Background

Nurses often face high levels of work-related stress that negatively impact their psychological well-being. In Thailand, these challenges are exacerbated by a persistent nursing shortage and heavy workloads, which have intensified in recent years, especially since the COVID-19 pandemic, due to increasing healthcare demands and high turnover rates [1]. These workforce pressures contribute to fatigue, reduced recovery time, and psychosocial distress among nurses. Studies have indicated that approximately 29–36% of nurses across different departments report feeling emotionally exhausted [2–5]. This emotional exhaustion often reflects a key indicator of burnout, a condition that encompasses multiple dimensions [6]. In particular, nurses experienced personal burnout (59.4%), work-related burnout (54.5%), and client-related burnout (42.3%) [7]. These outcomes are primarily driven by the demanding nature of nursing work, which includes long shifts, irregular hours, heavy patient care responsibilities, and inadequate compensation [8, 9]. Such conditions are also associated with poor sleep quality [10], unhealthy lifestyle behaviors, including increased consumption of junk food and emotional eating [11].

Emotional eating is defined as consuming food in response to emotional changes, especially negative emotions such as sadness, anxiety, or stress [12]. Among nurses, this behavior often emerges as a coping mechanism to alleviate emotional distress or improve mood [13]. However, frequent emotional eating, especially of high-calorie or low-nutrient food, increases the risk of weight gain and obesity [13]. As a result, nurses are considered a high-risk group for developing chronic non-communicable diseases, making emotional eating not only an individual health concern but also an occupational health issue that warrants greater attention [6]. In addition, psychosocial factors such as anxiety, depression, sleep disturbances, low self-control, and stressful life events have been strongly associated with emotional eating [14–16], further compounding the risks faced.

These psychosocial factors overlap considerably with burnout, which has also been linked to unhealthy dietary behavior, including emotional eating and poor dietary quality [6]. Recent international studies have explored the relationship between emotional eating and burnout in healthcare providers. Utter and McCray [17] found that poor diet quality was significantly associated with higher levels of burnout, suggesting that promoting healthier dietary behaviors may help mitigate burnout in clinical staff. Similarly, Chui and Bryant (2019) [18] identified a relationship between burnout and unhealthy eating behaviors among university staff in the United Kingdom, particularly an increase in consumption of fast food. These studies highlight the interplay between work stress, emotional exhaustion, and dietary choices in high-stress professional environments.

Despite these important contributions, there is limited research from Thailand that explores how psychosocial factors influence emotional eating, specifically among nurses. Cultural norms, healthcare system pressures, and differing workplace structures may influence both the experience of burnout and coping mechanisms, such as emotional eating [19]. Therefore, this study aims to examine the psychosocial factors associated with emotional eating in Thai nurses. The results may inform the development of effective interventions to promote healthier eating behaviors and improve both mental and physical health among this workforce. These insights are particularly significant, as they can inform organizational policies and health promotion programs, ultimately contributing to a more resilient and productive nursing workforce and supporting the delivery of safe, high-quality health care services.

## Methods

### Study design and setting

This study employed a cross-sectional design to examine the association between psychosocial factors and emotional eating among nurses in Thailand. Eligible participants were registered nurses of any gender, currently working full-time in public or private hospitals across Thailand. Data were collected between January and May 2025 using a self-administered online survey distributed via Google Forms. Once a participant accessed the electronic (online) survey, the first webpage contained an information sheet that explained the study’s purpose, ensured confidentiality, and affirmed the voluntary nature of participation. The cover letter also advised nurses that refusal to participate in the study would in no way jeopardize their employment status. The study was approved by the Chulabhorn Royal Academy Institutional Review Board (EC no.051/2567). All procedures were performed in accordance with the Declaration of Helsinki, ICH Guidelines for Good Clinical Practice, and other International Guidelines for Human Research protection.

### Sample

Participants were recruited using convenience sampling through social media platforms, professional nursing networks, and institutional outreach channels, such as LINE groups and Facebook pages. Researchers promoted and recruited only nurses who were eligible to complete the survey. The first screening question also verified participants’ professional status, allowing only those who identified as registered nurses currently practicing in Thailand to proceed with the survey. Before beginning the survey, all participants were required to read an electronic information sheet and provide informed consent by selecting the “Agree” option. To prevent duplicate submissions, researchers configured the Google Form system to ensure that participants could submit the survey only once. To improve the response rate and achieve the target sample size, the researchers promoted the survey through nursing-specific LINE groups, with the assistance of colleagues from various hospitals. Recruitment posts were updated every two weeks until the target number of participants was reached.

The required sample size was determined using the G*Power formula for a multiple linear regression model. The parameters set for the analysis included a power (1-β) of 0.80, a significance level (α) of 0.05, and an effect size (f²) of 0.15, the minimum sample size was calculated to be 153 participants. To allow for an anticipated 10% nonresponse rate, the final target sample was increased to 168 nurses.

## Measurement

### Demographic survey and workplace information

Data included age, gender, marital status, income sufficiency, chronic illness, smoking and alcohol use, exercise habits, BMI, hospital type, unit, years of experience, and working hours.

### Psychosocial factors

All instruments were administered in Thai. Tools originally developed in English were translated into Thai and reviewed for content accuracy through a translation and back-translation process conducted by bilingual nursing faculty. All measures used in this study are internationally standardized and widely validated instruments. The Thai versions employed were previously translated and culturally adapted [20, 21]; therefore, additional validation in this study was not required. Sleep quality was measured with the Jenkins Sleep Questionnaire (JSQ) [22], which includes four items evaluating sleep disturbances over the past month. Each item is rated on a 6-point scale ranging from 0 (no days) to 5 (22–28 days), with total scores ranging from 0 to 20. A score of 12 or higher indicates significant sleep problems. The Cronbach’s alpha coefficient reported for the JSQ was 0.79 [22]. Burnout was evaluated with the 19-item Copenhagen Burnout Inventory (CBI) [23]: personal burnout (6 items; α = 0.87), work-related burnout (7 items; α = 0.87), and client-related burnout (6 items; α = 0.85). Mean scores below 50 indicate low burnout, while scores of 51 or above indicate high burnout. Anxiety and depression were measured using the Thai versions of the Generalized Anxiety Disorder-7 (GAD-7) and the Patient Health Questionnaire-9 (PHQ-9) [21], both developed and validated by the Faculty of Medicine, Ramathibodi Hospital, Mahidol University. Total scores for the GAD-7 range from 0 to 21, and are interpreted as follows: 0–9 = mild anxiety, 10–14 = moderate anxiety, and 15–21 = severe anxiety. The Cronbach’s alpha coefficient reported for the GAD-7 was 0.92 [21]. The PHQ-9 ranges from 0 to 27, with interpretation as follows: less than 7 = no depression, 7–12 = mild depression, 13–18 = moderate depression, and 19 or above = severe depression. The Cronbach’s alpha reported for the PHQ-9 was 0.79 [20].

### Emotional eating

The Emotional Eating Questionnaire [24] includes 10 items assessing eating in response to emotions over the past two weeks. Scores range from 0 to 30, higher scores indicate a greater risk of emotional eating [25].

Three experts assessed content validity, and revisions were made accordingly. The finalized questionnaire was pilot-tested with 30 nurses with similar characteristics to the target population. The resulting reliability coefficients were as follows: JSQ (α = 0.81), CBI (α = 0.92, 0.81, 0.86 for personal burnout, work related burnout and client burnout, respectively), GAD-7 (α = 0.89), PHQ-9 (α = 0.90), and Emotional Eating Questionnaire (α = 0.89), indicating acceptable internal consistency across all measures.

### Data analysis

Data were analyzed using SPSS version 26.0 (IBM, Chicago, IL, USA), with the level of statistical significance at p-value *<* 0.05. Descriptive statistics, including frequency, percentage, mean, and standard deviation, were used to summarize demographics. Prior to inferential testing, the assumptions of linearity, independence, normality, and homoscedasticity were examined and found to be acceptable. Multicollinearity was also evaluated using tolerance and the variance inflation factor, which were within acceptable limits. Multiple linear regression was conducted to identify significant psychosocial predictors of emotional eating, after adjusting for sociodemographic factors.

## Results

### Characteristics of study participants

A total of 168 nurses (10 males and 158 females) were included in the analytic sample, with a mean age of 36.23 ± 7.45 years. The study achieved a 100% response rate. Most participants were single (90, 53.60%) and reported having sufficient income (131, 78.00%). A total of 44 nurses (26.20%) reported having chronic diseases, with mean BMI of 24.51 ± 4.68 kg/m^2^. None of the participants were current smokers, 45 (26.80%) reported not consuming alcohol, and the majority (121, 72.00%) engaged in regular exercise. Most participants worked in public hospitals (126, 75.00%). Regarding work unit, the largest proportion worked in the Emergency Room (ER) (42, 25.00%), followed by Out-patient Department (OPD) (38, 22.60%) and the In-patient Department (IPD) (22, 13.10%). While the remaining participants worked in other units such as the Labor Room (LR), Operating Room (OR), and the Hemodialysis unit (45, 26.80%). On average, participants had 13.13 ± 10.85 years of work experience, with a mean working time of 11.22 ± 3.42 h per day, and 5.50 ± 0.73 days per week (Table 1).

**Table 1:** Sociodemographic characteristics of the participants (*n* = 168)

	*N* (%)	
Age (years), mean *±* SD	36.23	*±* 7.45
Female	158	(94.00)
Monthly income (sufficiency)	131	(78.00)
Marital status		
Single	90	(53.60)
Married	69	(41.10)
Divorced/widowed	9	(5.40)
Current smokers (no)	168	(100.00)
Alcohol drinkers (no)	45	(26.80)
Exercise habits (yes)	121	(72.00)
BMI, mean *±* SD	24.51	*±* 4.68
Chronic diseases	44	(26.20)
Hospital type		
Public hospital	126	(75.00)
Private hospital	42	(25.00)
Hospital unit		
OPD	38	(22.60)
IPD	22	(13.10)
ICU	21	(12.50)
ER	42	(25.00)
Others (LR, OR, Hemodialysis unit, Primary hospital)	45	(26.80)
Years of experience, mean *±* SD	13.13	*±* 10.85
Working hours (per day), mean *±* SD	11.22	*±* 3.42
Working day (per week), mean *±* SD	5.50	*±* 0.73

BMI: body mass index; ER: emergency room; ICU; intensive care unit; IPD: in-patient department; LR: labor room; OPD: out-patient department; OR: operating room; SD: standard deviation

### Distribution of scores on the psychosocial factors and emotional eating among nurses

As shown in Table 2, the mean sleep score was 3.83 *±* 3.21, indicating that nurses did not report sleep disturbances. Burnout levels were low, with personal burnout highest at 35.67 *±* 19.48, followed by work-related burnout at 33.92 *±* 14.66 and client-related burnout at 31.35 *±* 17.97. Anxiety and depression levels were mild, with a mean score of 3.98 *±* 3.68 on the GAD-7 and 4.68 *±* 4.57 on the PHQ-9. Emotional eating was measured using the Emotional Eating Questionnaire, yielding a mean score of 7.42 *±* 4.95.

**Table 2:** Distribution of scores on the psychosocial factors and emotional eating among nurses (*n* = 168)

	mean	*±* SD
Sleep problems	3.83	*±* 3.21
Personal burnout	35.69	*±* 19.48
Work burnout	33.92	*±* 14.66
Client burnout	31.35	*±* 17.97
GAD-7	3.98	*±* 3.68
PHQ-9	4.68	*±* 4.57
Emotional Eating	7.42	*±* 4.95

GAD-7: Generalized Anxiety Disorder; PHQ-9: Patient Health Questionnaire-9; SD: standard deviation

### Multiple linear regression analysis predicting emotional eating among nurses

The multiple linear regression model explained 33.0% of the variance in emotional eating among nurses (adjusted R^2^ = 0.330) (Table 3). Client-related burnout (B = 0.054, *p* = 0.007), anxiety (B = 0.526, *p* < 0.001), and depression (B = 0.233, *p* = 0.034) were significant positive predictors of emotional eating, indicating that higher levels of psychological distress related to patient care were associated with greater emotional eating. Interestingly, personal-related burnout demonstrated a significant negative association with emotional eating (B = -0.057, *p* = 0.009). Sleep problems and work-related burnout were not significant predictors.

**Table 3:** Multiple linear regression analysis predicting emotional eating among nurses

Predictor variables	B (Unstandardized Coefficient)	SE B	β (Standardized Coefficient)	t	*p*-value
(Constant)	4.017	0.932		4.308	0.000
Sleep problems	0.164	0.117	0.106	1.402	0.163
Personal burnout	-0.057	0.022	-0.223	-2.635	0.009
Work burnout	-0.003	0.023	-0.008	-0.115	0.909
Client burnout	0.054	0.020	0.196	2.727	0.007
GAD-7	0.526	0.127	0.390	4.142	< 0.001
PHQ-9	0.233	0.109	0.215	2.136	0.034

GAD-7: Generalized Anxiety Disorder; PHQ-9: Patient Health Questionnaire-9

R^2^ = 0.354, Adjusted R^2^ = 0.330

## Discussions

This study examined the association between psychosocial factors and emotional eating among nurses in Thailand. Our findings found the positive associations between anxiety, depression, and emotional eating are consistent with prior research, indicating that emotional distress [12, 26, 27] is a major determinant of maladaptive eating behaviors. Nurses experiencing higher levels of psychological distress may use food consumption as a coping strategy [13], which is aligned with affect-regulation models of emotional eating [28]. Evidence suggests that structured programs such as mindfulness training and resilience workshops are effective in reducing emotional eating and enhancing coping capacity [29, 30]. These findings underscore the importance of providing psychological support and stress management interventions to mitigate emotional eating among nurses.

The different domains of burnout may exert distinct influences on eating behaviors. Personal burnout reflects chronic fatigue and stress in one’s private life, which may reduce energy and motivation. When personal burnout is high, individuals may disengage in coping responses, including eating. Workplace and cultural factors, such as limited break times, reliance on convenience foods, and the culture of shared eating during stressful shifts, may also further reinforce this behavior [31]. Client-related burnout, reflecting stress and exhaustion from direct patient interactions [8], may heighten vulnerability to emotional eating as a compensatory behavior. Studies indicate that burnout is associated with more frequent stress-driven eating habits, which involve using food as a coping mechanism [6], such as intake consumption of unhealthy and high-calorie foods [32]. In contrast, work-related burnout was not significantly associated with emotional eating in this study. This finding suggests that overall work-related stress and exhaustion may not directly translate into maladaptive eating behaviors among nurses [33]. One possible explanation is that work-related burnout reflects general occupational demands, which nurses may manage through adaptive coping strategies rather than food consumption [34]. 

Contrary to expectations, sleep problems were not significantly associated with emotional eating. Although poor sleep has been linked to dysregulated appetite and increased emotional activity [35], it is possible that the relatively young age and adaptive coping strategies of the sample may have mitigated this relationship [36]. Alternatively, the effect of sleep may be mediated through anxiety and depression [37, 38], which are recognized as more direct contributors to maladaptive eating.

Overall, these findings highlight the complex interplay between psychosocial factors and emotional eating in nurses. Given the high prevalence of burnout and psychological distress in the nursing population [2, 7], targeted interventions aimed at reducing client-related stress, enhancing resilience, coping strategies, and addressing mental health symptoms may be beneficial [29, 36, 39]. Moreover, healthcare systems should integrate psychosocial health promotion into organizational policies and workplace design. Such integration may include developing institutional programs that monitor and reduce burnout, provide mental health screening, and promote healthy coping strategies such as mindfulness and emotional regulation training [40–42]. Furthermore, improving nurse-to-patient ratios, providing supportive supervision, and ensuring access to nutritious meals at work may reduce emotional eating and enhance overall well-being among nurses [43, 44]. Future longitudinal study research is needed to determine whether reducing burnout and psychological distress can directly decrease emotional eating, and to identify potential moderating factors, such as coping style or support systems, that influence whether different forms of burnout contribute to emotional eating in healthcare professionals.

### Limitation

This study has several limitations. First, the relatively small sample size may limit the generalizability of the finding. Second, the cross-sectional design may be subject to self-report bias, which could affect the accuracy of the measured variables. Despite these limitations, this study has notable strengths. The sample encompassed nurses from all regions of Thailand and included various departments across both public and private hospitals. This broad coverage helps reduce sampling bias and enhances representativeness of the findings, providing a more comprehensive understanding of the psychosocial factors associated with emotional eating among Thai nurses.

## Conclusions

The findings suggest that personal burnout, client-related burnout, anxiety, and depression were significant predictors of emotional eating. Notably, client-related burnout, anxiety, and depression were positively associated with emotional eating. Therefore, interventions aimed at reducing burnout, particularly client-related burnout, as well as programs targeting anxiety and depressive symptoms, are warranted to support the well-being of professional nurses.

## Data Availability

The datasets generated and analyzed during the current study are available from the corresponding author on reasonable request.

## Abbreviations

CBI: Copenhagen Burnout Inventory
GAD-7: Generalized Anxiety Disorder-7
JSQ: Jenkins Sleep Questionnaire
PHQ-9: Patient Health Questionnaire-9
